# P-399. Prevalence of gastrointestinal tract colonization by carbapenem-resistant Enterobacterales and vancomycin-resistant *Enterococcus faecium* in hematological patients at a referral hospital in Mexico City

**DOI:** 10.1093/ofid/ofae631.600

**Published:** 2025-01-29

**Authors:** Ana Fernanda Ramos Menchelli, María Fernanda González Lara, Bernardo Martínez Guerra, Oscar Fernández, Edgar Daniel Centeno Matus, Luis Fernando Xancal Salvador, Roberta Demichelis Gómez, Eucario León Rodríguez

**Affiliations:** Instituto Nacional de Ciencias Médicas y Nutrición Salvador Zubirán, Mexico City, Distrito Federal, Mexico; Instituto Nacional de Ciencias Médicas y Nutrición Salvador Zubirán, Mexico City, Distrito Federal, Mexico; Instituto Nacional de Ciencias Médicas y Nutrición Salvador Zubirán, Mexico City, Distrito Federal, Mexico; Instituto Nacional de Ciencias Médicas y Nutrición Salvador Zubirán, Mexico City, Distrito Federal, Mexico; Instituto Nacional de Ciencias Médicas y Nutrición Salvador Zubirán, Mexico City, Distrito Federal, Mexico; Instituto Nacional de Ciencias Médicas y Nutrición Salvador Zubirán, Mexico City, Distrito Federal, Mexico; Instituto Nacional de Ciencias Médicas y Nutrición Salvador Zubirán, Mexico City, Distrito Federal, Mexico; Instituto Nacional de Ciencias Médicas y Nutrición Salvador Zubirán, Mexico City, Distrito Federal, Mexico

## Abstract

**Background:**

The prevalence of carbapenem-resistant enterobacteria (CRE) in patients with hematological neoplasias varies due to local epidemiology, antimicrobial prophylaxis and types of hematopoietic stem cell transplants (HSCT). Colonization by CRE is the main risk factor for infection, which carries a mortality up to 65%. This study evaluated CRE colonization in patients with acute leukemia (AL) and HSCT) within 28 days. Secondary goals included describing CRE bloodstream infections (BSI), colonization by Vancomycin-Resistant Enterococci (VRE), ESBL, and quinolone-resistant (QR) microorganisms, risk factors for CRE, and 28-day mortality.

**Table 1.**
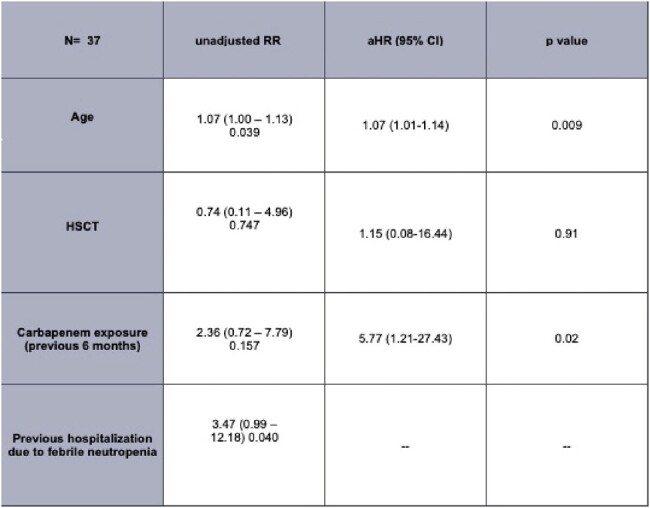
Risk factors for CRE colonization among patients with acute leukemia and hematopoietic stem cell transplant RR: Relative risk, aHR: Adjusted hazard ratio, allo HSCT: Allogeneic hematopoietic stem cell transplant

**Methods:**

This was a prospective cohort from March to October 2023 at a referral hospital in Mexico City. We included consenting adults with acute leukemia (AL) receiving induction chemotherapy and HSCT. Patients with prior 90-day CRE colonization or infection were excluded. Weekly stool screening, using the adapted CDC method, was performed throughout hospitalization, with a follow-up period of 28 days or until discharge or death. CRE were identified by MALDITOF-MS (Bruker), susceptibility testing done by Vitek2 (Biomérieux), using CDC breakpoints. Descriptive analysis was done for CRE colonization and mortality. A multivariate analysis for CRE colonization through logistic regression was done.

**Results:**

Among 37 patients included, a CRE colonization prevalence of 21.8% was found, of which, 12.5% developed BSI compared to 3.5% of non-colonized individuals. Additionally, colonization rates for VRE, ESBL, and QR microorganisms were 5.4%, 35.1%, and 32.4%, respectively. Independent factors related to CRE colonization were older age and prior 6-month carbapenem use. 28-day mortality was 10.8%, with no differences between colonized and non-colonized groups. VRE colonization was significantly associated with VRE infection (p=0.04). CRE colonization resulted in increased 90-day mortality (50% vs. 10.3%, p=0.011).

**Conclusion:**

The prevalence of CRE colonization in patients with AL or HSCT was 21.8%. It was associated with older age and prior carbapenem use and resulted in increased 90-day mortality. Colonization by ESBL and QR were frequent.

**Disclosures:**

**All Authors**: No reported disclosures

